# Persistent disparities in antiretroviral treatment (ART) coverage and virological suppression across Europe, 2004 to 2015

**DOI:** 10.2807/1560-7917.ES.2018.23.21.1700382

**Published:** 2018-05-24

**Authors:** Kamilla Laut, Leah Shepherd, Roxana Radoi, Igor Karpov, Milosz Parczewski, Cristina Mussini, Fernando Maltez, Marcelo Losso, Nikoloz Chkhartishvili, Hila Elinav, Helen Kovari, Anders Blaxhult, Robert Zangerle, Tatiana Trofimova, Malgorzata Inglot, Kai Zilmer, Elena Kuzovatova, Thérèse Staub, Dorthe Raben, Jens Lundgren, Amanda Mocroft, Ole Kirk, on behalf of the EuroSIDA Study Group

**Affiliations:** 1 CHIP, Centre of Excellence for Health, Immunity and Infections, Department of Infectious Diseases, Rigshospitalet, University of Copenhagen, Copenhagen, Denmark; 2 Centre for Clinical Research, Epidemiology, Modelling and Evaluation (CREME), Institute for Global Health, University College London, London, United Kingdom; 3 Spitalul Clinic de Boli Infectioase si Tropical, “Dr. Victor Babes”, Bucharest, Romania; 4 Department of Infectious Diseases, Belarus State Medical University, Minsk, Belarus; 5 Department of Infectious, Tropical Diseases and Immune Deficiency, Pomeranian Medical University, Szczecin, Szczecin, Poland; 6 Clinica delle Malattie Infettive e Tropicali, Università degli Studi di Modena e Reggio Emilia, Modena, Italy; 7 Hospital de Curry Cabral, Serviço de Doenças Infecciosas, Lisbon, Portugal; 8 Coordinación de Investigación Clinica Académica en Latinoamérica, Buenos Aires, Argentina; 9 Infectious Diseases, AIDS and Clinical Immunology Research Center, Tbilisi, Georgia; 10 Department of Clinical Microbiology and Infectious Diseases, Hadassah Hospital, Jerusalem, Israel; 11 Division of Infectious Diseases and Hospital Epidemiology, University Hospital Zurich, University of Zurich, Zurich, Switzerland; 12 Department of Infectious Diseases, Venhälsan, Södersjukhuset, Stockholm, Sweden; 13 Medical University of Innsbruck, Department of Dermatology, Venereology and Allergology, Innsbruck, Austria; 14 Novgorod Centre for AIDS Prevention and Control, Velikij Novgorod, Russia; 15 Medical University Wroclaw, Wroclaw, Poland; 16 Centre of Infectious Diseases, West-Tallin Central Hospital, Tallin, Estonia; 17 Nizhny Novgorod Scientific and Research Institute of Epidemiology and Microbiology named after Academician I.N. Blokhina, Nizhny Novgorod, Russia; 18 Centre Hospitalier de Luxembourg, Service des Maladies Infectieuses, Luxembourg; 19 The members of the EuroSIDA Study Group are acknowledged at the end of the article

**Keywords:** HIV infection, Epidemiology, Surveillance, Continuity of patient care, Treatment outcome, Europe

## Abstract

Background: Direct comparisons between countries in core HIV care parameters are often hampered by differences in data collection. Aim: Within the EuroSIDA study, we compared levels of antiretroviral treatment (ART) coverage and virological suppression (HIV RNA < 500 copies/mL) across Europe and explored temporal trends. Methods: In three cross-sectional analyses in 2004–05, 2009–10 and 2014–15, we assessed country-specific percentages of ART coverage and virological suppression among those on ART. Temporal changes were analysed using logistic regression. Results: Overall, the percentage of people on ART increased from 2004–05 (67.8%) to 2014–15 (78.2%), as did the percentage among those on ART who were virologically suppressed (75.2% in 2004–05, 87.7% in 2014–15). However, the rate of improvement over time varied significantly between regions (p < 0.01). In 2014–15, six of 34 countries had both ART coverage and virological suppression of above 90% among those on ART. The pattern varied substantially across clinics within countries, with ART coverage ranging from 61.9% to 97.0% and virological suppression from 32.2% to 100%. Compared with Western Europe (as defined in this study), patients in other regions were less likely to be virologically suppressed in 2014–15, with the lowest odds of suppression (adjusted odds ratio = 0.16; 95% confidence interval (CI): 0.13–0.21) in Eastern Europe. Conclusions: Despite overall improvements over a decade, we found persistent disparities in country-specific estimates of ART coverage and virological suppression. Underlying reasons for this variation warrant further analysis to identify a best practice and benchmark HIV care across EuroSIDA.

## Background

It is documented that large health inequalities exist across Europe among people living with HIV (PLHIV) as well as for other diseases [1-4]. In recent years, comparing and characterising differences in healthcare between countries has received growing interest and has become a central component of informing and targeting health policies. Since it was first introduced, the HIV care continuum has been widely adopted as a tool to benchmark the quality of HIV care [2,5,6], and there are several examples of national and local HIV care continua, including a number of European countries [7-17]. The 90–90–90 targets, launched by the Joint United Nations Programme on HIV/AIDS (UNAIDS) in 2014, aim to set goals for improving the HIV continuum of care from diagnosis to virological suppression [18]. More specifically, they state that at least 90% of PLHIV should be aware of their status, at least 90% of people diagnosed with HIV should receive antiretroviral therapy (ART) and at least 90% of those should be virologically suppressed. If these targets are reached by 2020, ending the AIDS epidemic by 2030 should be within reach [18]. It is clear that some countries are close to reaching the targets while others still have a long way to go [2,19,20]. However, in the absence of common definitions for the different steps of the care continuum, country-to-country comparisons have proven difficult [2,6,20-22]. Furthermore, differences in the data sources used to construct the continua of care further complicate international comparisons.

Previous studies comparing the HIV care continuum across countries have been limited by such differences in data collection [19,20], and there is currently a lack of studies with access to internationally comparable data. EuroSIDA has a unique set-up which allows direct comparisons of data between countries. The aims of this study were to characterise country-specific levels of antiretroviral coverage and ART-induced HIV RNA suppression within the EuroSIDA study, and to monitor temporal trends.

## Methods

### Patients

The EuroSIDA cohort study includes data from (as at May 2017) 23,043 PLHIV 16 years or older and enrolled in HIV care at 100 HIV outpatient facilities in 33 countries across continental Europe as well as Israel and Argentina. Details about the study have been published elsewhere [23]. In brief, EuroSIDA collects patient information that is routinely recorded at the collaborating sites. Data are reported to EuroSIDA on standardised data collection forms, first at enrolment into the study, and thereafter at 6-month intervals. At each data collection, demographic and clinical data, including all CD4^+^ T-cell counts and HIV RNA measurements, data about ongoing ART and reasons for stopping or switching treatment, date of diagnosis of any AIDS- or non-AIDS-defining illness, and information about cause of death is recorded. Data quality assurance includes site visits with source verification of all major clinical events and monitoring data from a random selection of patients followed at each site. EuroSIDA is an open cohort, and in the period from 2004 to 2016, additional patients and sites were recruited in four recruitment waves in 2005 (n = 2,500), 2008 (n = 2,500), 2012 (n = 2,500) and 2014–2016 (n = 4,000). Details about loss to follow-up within EuroSIDA have previously been reported [24].

### Definitions

EuroSIDA participants were followed from recruitment into EuroSIDA until the latest CD4^+^ T-cell measurement, HIV RNA-measurement, date of most recent visit to clinic, or death. In three a priori defined time periods, a person was considered ‘in care’ if their first EuroSIDA visit occurred before the end of the time period assessed and their latest recorded visit or CD4^+^ T-cell count or HIV RNA measurement occurred after the beginning of the time period assessed. People were assessed for being ‘in care’, ‘on ART’ or ‘virologically suppressed’ at the latest of a clinic visit, a CD4^+^ T-cell or an HIV RNA-measurement in each time period. If neither was available, the midpoint of the period was used. A 12 months window was allowed before the assessment date. This means that if a clinic visit date or a CD4^+^ T-cell count (and date) was available within the period assessed, an HIV RNA measurement within 12 months prior to that date could be included in analyses. The outcomes of interest were country-specific percentages of ‘on ART’, defined as the number of people receiving ART among those in care, and of ‘virologically suppressed’, defined as the number of people with HIV RNA (most recent measurement) below 500 copies/mL among those on ART. ART was defined as receiving at least three antiretroviral drugs from any class. The cut-off of 500 copies/mL was chosen as not all countries have access to assays with a lower limit of detection of 50 copies/mL. A person in care but with no available HIV RNA measurement within the time period assessed, was considered virologically unsuppressed (missing = failure). People who were followed in EuroSIDA during more than one of the time periods assessed could contribute data to more than one time period. In sensitivity analyses, we assessed the influence of recent recruitment into EuroSIDA and of using an HIV RNA detection limit of 50 copies/mL.

### Statistical analyses

In repeated cross-sectional analyses, we compared country- and region-specific percentages of people on ART and virologically suppressed in three time periods: 1 January 2004 to 31 December 2005, 1 January 2009 to 31 December 2010, and 1 January 2014 to 31 December 2015. Country-level estimates were based on pooled results from active EuroSIDA clinics in the country during each time period, and were grouped into regions as listed in Table 1. Because this study had a European focus, data from Argentinian clinics were not included in analyses. Israel is part of the World Health Organisation European Region and was included in the Southern European region, as per EuroSIDA tradition. We chose not to report individual country estimates based on fewer than 30 people.

**Table 1:** Definition of European regions for his study, EuroSIDA study, 2004–2015 (n = 35 countries)

Western Europe	Southern Europe	Northern Europe	East Central Europe	Eastern Europe
Austria	Greece	Denmark	Bosnia-Herzegovina^a^	Belarus
Belgium	Israel	Finland	Croatia^a^	Estonia
France	Italy	Iceland^a^	Czech Republic	Georgia^a^
Germany	Portugal	Ireland	Hungary	Latvia
Luxembourg	Spain	The Netherlands	Poland	Lithuania
Switzerland		Norway	Romania	Russia
		Sweden	Serbia	Ukraine
		United Kingdom	Slovakia^b^	
			Slovenia^a^	

^a^ Countries included only in the 2014–15 cohort.

^b^ Countries included only in the 2004–05 cohort.

Trends over calendar time were analysed using logistic regression with generalised estimating equations, accounting for repeated measurements and adjusting for basic patient characteristics that were thought to vary significantly between regions over time, including current age, sex, mode of infection, CD4^+^ T-cells at entry into EuroSIDA, and current hepatitis B and hepatitis C status. Formal tests for interaction between regions and time were performed.

## Results

### Patient and clinic characteristics

Table 2 shows the characteristics of patients and clinics included in analyses during the three time periods. 12,825 people were under follow-up in the 2014­–15-cohort in 105 clinics in 34 countries. Of them 10,034 (78.2%) patients were on ART and of those, 8,803 (87.7%) patients were virologically suppressed. A total of 758 people on ART (7.5%) did not have HIV RNA measurements in the 2014–15 study period. The percentage of people without available HIV RNA measurement were equally distributed across Southern Europe (6.7%), Northern Europe (9.5%), East Central Europe (9.5%), and Eastern Europe (11.6%), but was lower in Western Europe (2.1%). The percentage without available HIV RNA measurement in 2014­–15 varied between 58.3% of those on ART in one country, to less than 1% in three countries.

**Table 2:** Characteristics of patients and clinics included in analyses during the three time periods, EuroSIDA study, 2004–05, 2009–10 and 2014–15

	2004–05		2009–10		2014–15	
Number of clinics included	96		100		105	
Total number of patients included	8,743		10,013		12,825	
Median number of patients per clinic, n (IQR)	74 (39–114)		90 (49–136)		108 (64–167)	
Age in years, median (IQR)	40.9 (35.6–47.8)		44.9 (37.8–51.8)		48.3 (39.9–54.7)	
	n	%	n	%	n	%
Sex						
Male	6,543	74.8	7,257	72.5	9,352	72.9
Female	2,200	25.2	2,756	27.5	3,473	27.1
Region of residence						
Western Europe	2,137	24.4	2,385	23.8	3,033	23.6
Southern Europe	2,586	29.6	2,420	24.2	2,957	23.1
Northern Europe	2,218	25.4	2,319	23.2	2,902	22.6
East Central Europe	975	11.2	1,409	14.1	1,934	15.1
Eastern Europe	827	9.5	1,480	14.8	1,999	15.6
Mode of infection						
MSM	3,692	42.2	4,143	41.4	4,772	37.2
PWID	2,053	23.5	2,087	20.8	3,363	26.2
Heterosexual	2,432	27.8	3,084	30.8	3,750	29.2
Other/unknown	566	6.5	699	7.0	940	7.3
Number of patients on ART, % of total	5,928	67.8	7,687	76.8	10,034	78.2
By mode of infection, % of risk group						
MSM	2,774	75.1	3,364	81.2	3,845	80.6
PWID	1,171	57.0	1,421	68.1	2,439	72.5
Heterosexual	1,560	64.1	2,347	76.1	2,997	79.9
Other/unknown	423	74.7	555	79.4	753	80.1
Patients not receiving ART with CD4^+^ T-cell count < 200 cells/mL, % of those not on ART	405	14.4	192	8.3	280	10.0
By region of residence, % of those not on ART in that region						
Western Europe	81	13.0	36	6.7	75	9.8
Southern Europe	92	14.4	32	7.0	44	5.5
Northern Europe	98	19.4	18	6.4	13	3.6
East Central Europe	66	19.4	20	6.7	34	11.8
Eastern Europe	68	9.6	86	11.4	114	20.0
Patients with missing information on HIV RNA among those on ART, % of total on ART	188	3.2	455	5.9	758	7.6
Patients with HIV RNA < 500 copies/mL among those on ART, % of total on ART	4,460	75.2	6,777	88.2	8,803	87.7
By mode of infection, % of risk group on ART						
MSM	2,136	77.0	3,107	92.4	3,523	91.6
PWID	861	73.5	1,135	79.9	1,976	81.0
Heterosexual	1,152	73.8	2,036	86.7	2,631	87.8
Other/unknown	311	73.5	499	89.9	673	89.4

ART: antiretroviral treatment; IQR: interquartile range; MSM: men who have sex with men; PWID: people who inject drugs.

The median age of the study population increased over the three time periods and was 48.3 years (interquartile range: 39.9–54.7) in 2014–15. Patients were predominantly male (72.9% in 2014–15) and throughout the study period, the predominant mode of infection was sex between men (37.2% in 2014–15), followed by heterosexual transmission (29.2%) and injection drug use (26.2%). As EuroSIDA included new clinics and patients over time, the number of people enrolled in East Central and Eastern Europe increased, changing the relative distribution between regions over time. Of the total 16,826 people included across all three time periods, 9,224 (54.8%) contributed data to more than one time period.

Overall, 280 patients (10.0%) of 2,791 who did not receive ART in 2014–15 had a CD4^+^ T-cell count below 200 cells/mL. This percentage was higher in in Eastern Europe (20.0%) than in the other regions (Western Europe 9.8%, Southern Europe 5.5%, Northern Europe 3.6%, East Central Europe 11.8%, p for difference across regions < 0.0001)

### Country-specific levels of ART coverage and virologically suppressed among those on ART

**Figure 1:**
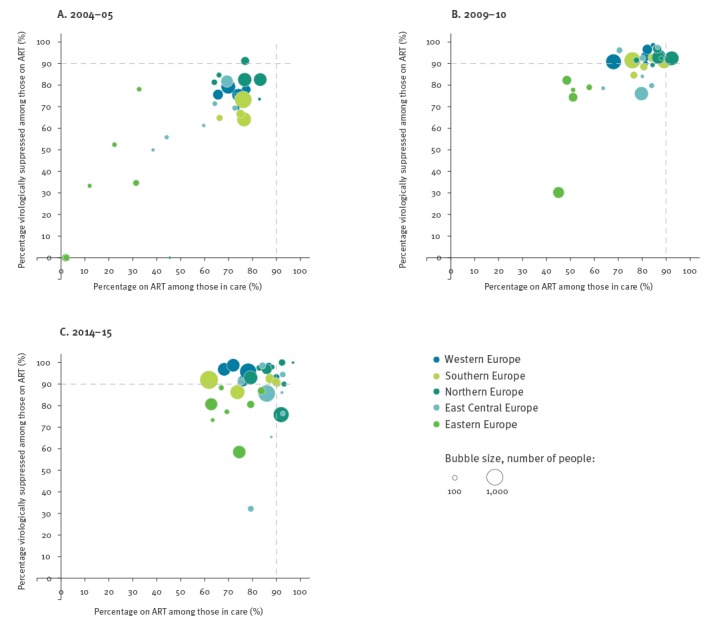
Country-specific estimates of the percentage of people on ART among those in care, and percentage of people virologically suppressed among those on ART, EuroSIDA study, 2004–05, 2009–10 and 2014–15

Figure 1 shows unadjusted country-level estimates of the percentage of people on ART among those in care and the percentage virologically suppressed among those on ART in the three time periods. Each country is represented by a bubble and the area of each bubble is proportional to the total number of people under follow-up in each country during each time period. Individual country estimates based on fewer than 30 people are not shown. In 2014–15 (Figure 1C) the highest-ranking country had an ART coverage of 97.0%, and all of those were virologically suppressed. The country with the lowest percentage on ART was a country in Southern Europe with 61.9% on ART among those in care, and the country with the lowest percentage virologically suppressed was a country in East Central Europe, where 32.2% of those on ART were virologically suppressed. Fourteen of 34 countries had levels of virological suppression among those on ART below 90%. In six of 34 countries, more than 90% were on ART and more than 90% were virologically suppressed among those on ART, while 11 of 34 countries reached neither target in 2014–15.

ART: antiretroviral treatment.

Each bubble represents a country and the area of the bubble is proportional to the number of people under follow-up in each country. Virological suppression was defined as HIV RNA < 500 copies/mL. The two dotted lines indicate 90% of those in care on ART (x-axis) and 90% virologically suppressed among those on ART (y-axis). Individual country estimates based on fewer than 30 people are not shown.

### Temporal trends

**Figure 2:**
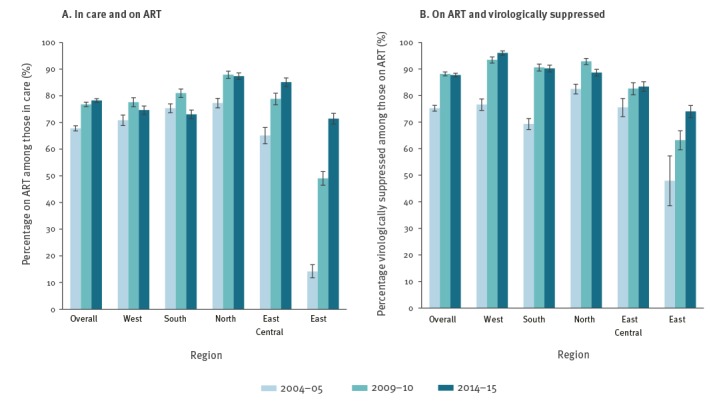
Temporal trends in unadjusted estimates of the percentage on ART among those in care and percentage virologically suppressed among those on ART by region and overall, EuroSIDA study, 2004–05, 2009–10 and 2014–15

**Figure 3:**
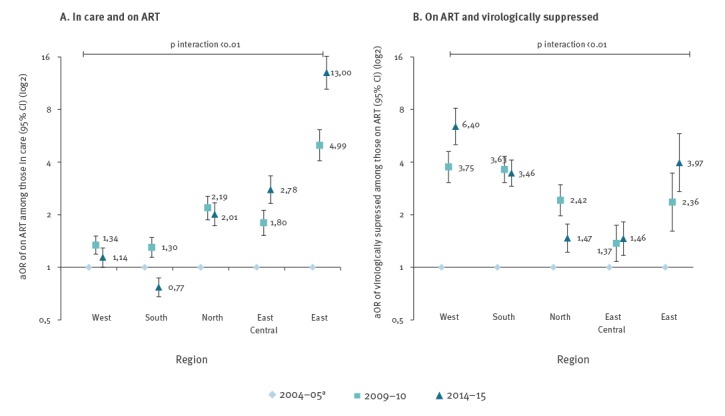
Adjusted odds ratio of being on ART among those in care and of being virologically suppressed among those on ART in 2009–10 and 2014–15 compared with 2004–05 by region, EuroSIDA study

ART: antiretroviral treatment; aOR: adjusted odds ratio; CI: confidence interval.

^a^ 2004–05 reference.

Figure 2A shows how crude estimates of the percentage of people on ART changed over time, overall and by region. Overall, the percentage of people receiving ART increased from 67.8% in 2004–05 to 78.2% in 2014–15. The level of ART coverage varied substantially between regions, but changed over time to become more consistent across regions (p < 0.01 for interaction, Figure 3A). For example, ART coverage was low in Eastern Europe in 2004–05, but people in this region were 13 times more likely to receive ART in 2014–15 compared with 2004–05 (adjusted odds ratio (aOR) on ART = 13.00; 95% confidence interval (CI): 10.46–16.15). In Western Europe, ART coverage in 2004–05 was higher and the odds of receiving ART increased correspondingly less over time (on ART 2014–15 vs 2004–05: aOR = 1.14; 95% CI: 1.00–1.29). While ART coverage increased between 2004–05 and 2009–10, levels seemed to reach a plateau in recent years in Northern Europe (on ART 2014–15 vs 2004–05: aOR = 2.01; 95% CI: 1.73–2.34) and declined slightly in Southern Europe (aOR = 0.77; 95% CI: 0.68–0.87).

ART: antiretroviral treatment.

Bars indicate 95% confidence intervals. Virological suppression was defined as HIV RNA < 500 copies/mL.

The crude percentage of people who were virologically suppressed among those on ART also increased over time (Figure 2B), and the change over time again varied between regions (p < 0.01 for interaction, Figure 3B). This ranged from a 46% increase in East Central Europe (virologically suppressed 2014­–15 vs 2004–05: aOR = 1.46; 95% CI: 1.17–1.82) and a 47% increase in Northern Europe (aOR = 1.47; 95% CI: 1.22–1.77), to a more than threefold increase in Southern Europe (aOR = 3.46; 95% CI: 2.91–4.11). The largest improvements over time were observed in Western Europe, where the odds of virological suppression were more than six times higher (aOR = 6.40; 95% CI: 5.03–8.14) in 2014–15 compared with 2004–05, and in Eastern Europe with a fourfold increase in the odds of virological suppression (aOR = 3.97; 95% CI: 2.71–5.82) over the decade.

Odds ratios are on a log2 scale. Virological suppression was defined as HIV RNA < 500 copies/mL. Adjusted for current age, sex, risk group, CD4^+^ T-cells at entry into EuroSIDA, current hepatitis B status, current hepatitis C status.

### Regional variability

**Figure 4:**
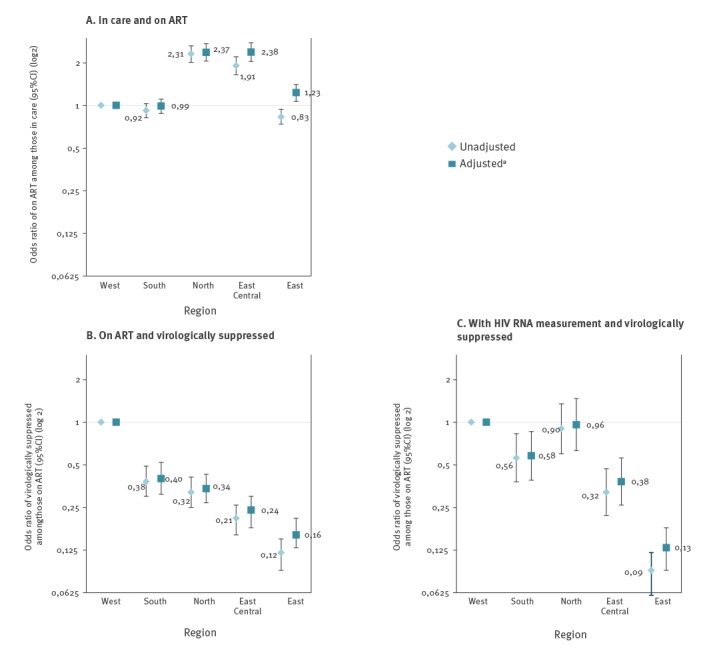
Adjusted odds ratio of being on ART among those in care, of being virologically suppressed among those on ART, and of being virologically suppressed among those with an available HIV RNA measurement, EuroSIDA study, 2014–2015 (n = 12,825)

Figure 4A shows the 2014–15 estimates of the unadjusted and adjusted odds of receiving ART across the five regions. Compared with Western Europe, people in Northern Europe (aOR on ART = 2.35; 95% CI: 2.04–2.70) and East Central Europe (aOR = 2.39; 95% CI: 2.05–2.78) were more likely to receive ART in both unadjusted and adjusted models. Conversely, in unadjusted estimates, people in Eastern Europe (OR = 0.83; 95% CI: 0.74–0.94) were less likely to receive ART, but after adjustment, the odds of being on ART were higher in Eastern compared with Western Europe (aOR = 1.23; 95% CI: 1.07–1.41). There was no evidence of differences in ART coverage between Western and Southern Europe (aOR = 1.07; 95% CI: 0.96–1.20).

ART: antiretroviral treatment.

Western Europe reference. Odds ratios are on a log2 scale. Virological suppression was defined as HIV RNA < 500 copies/mL. Total number of people excluded from analysis in panel C: n = 758 (7.6%).

^a^ Adjusted for current age, sex, risk group, CD4^+^ T-cells at entry into EuroSIDA, current hepatitis B status, current hepatitis status.

Figure 4B shows the 2014–15 estimates of the unadjusted and adjusted odds of virological suppression among those on ART, comparing across regions. Compared with Western Europe, patients in all other regions were less likely to be virologically suppressed in 2014–15.

### Sensitivity analyses

In sensitivity analyses, differences in the availability of HIV RNA measurements seemed to explain some of the differences between regions (Figure 4C). Thus, the lower odds of virological suppression observed in Northern Europe disappeared when excluding people with missing HIV RNA measurements in 2014–15 (aOR = 0.96; 95% CI: 0.63–1.47) and were attenuated in East Central and Southern Europe. Conversely, the lower odds of virological suppression remained for people in Eastern Europe, even when excluding those with missing HIV RNA measurements (aOR = 0.13; 95% CI: 0.09–0.18).

EuroSIDA is an open cohort, and the latest enrolment wave was 2014–16. To account for differences in ART coverage and virological suppression among these newly enrolled patients, we performed sensitivity analyses excluding 2,893 patients recruited into EuroSIDA in 2014–15. Excluding these patients yielded overall comparable results, although unadjusted point estimates for some individual countries did change (data not shown).

## Discussion

In this study we provide a picture of variation in ART coverage and virological suppression in a large number of clinics across 34 countries in Europe including Israel, and describe trends over the last decade. We found that overall, ART coverage increased between 2004–05 and 2014–15, as did the percentage of people with suppressed viral load among those on ART, but these overall improvements covered very large differences in country-specific estimates and trends in ART uptake and virological suppression. We also found that in 2014–15, 14 of 34 countries did not reach the goal of virological suppression in at least 90% of those on ART among those persons included in EuroSIDA.

Our data help illustrate that countries may have very different challenges in improving outcomes along the continuum of care. Following the findings of the Strategic Timing of Antiretroviral Therapy (START) study [25] and the subsequent adaptation of clinical guidelines to initiate ART in any person regardless of CD4^+^ T-cell count [26,27], we may expect ART coverage to increase in the years to come, although not all countries have adapted the recommendations in national clinical guidelines. Expanding the use of ART will require substantial funding, especially in countries where ART coverage is low. Further, although countries in Eastern Europe experienced the largest improvements in ART coverage over time, some of these countries also have the highest burden of HIV and are likely to face a range of challenges with scaling up their ART programmes.

We chose to include patients with missing HIV RNA measurements in our analyses, while others have attempted to statistically account for [9,28,29] or exclude [7] those with missing values. Our approach may underestimate the true number of people with suppressed HIV RNA, whereas excluding all with missing HIV RNA measurements is likely to overestimate the percentage of people successfully managed. HIV RNA measurements may be missing for people who receive ART for many reasons, including irregular clinic attendance, patient refusal, poor access or limited resources for laboratory monitoring. Measurements may also be missing at clinics with reporting delays or at sites that have reported incomplete data. We were not able to distinguish between these different causes which may have very different implications for the individual patient. However, it is worth noting that the observed differences in levels of virological suppression were not explained by differences in the availability of HIV RNA measurements. Also, our window for including an HIV RNA measurement was broad, and we find it reasonable to assume that a patient without available HIV RNA measurement within a 2-year period is not successfully managed and may signal a potential opportunity for intervention or a missed opportunity for retaining a patient in care, in particular in the 2014–15 study period. Furthermore, a lack of data may in itself be a sign of possible gaps in performance and may thus equally be a sign of an opportunity for intervention [30].

Our definition of virological suppression was based on a single HIV RNA measurement below 500 copies/mL. In a previous study we showed that current viral load was as good as repeated HIV RNA measurements to evaluate quality of ART care [31]. This is also in line with strategies recommended by the European Centre for Disease Prevention and Control (ECDC) and UNAIDS [2,18]. However, some may consider this a liberal definition of virological suppression and may argue that confirmed measurements are more reliable. If this is true, we expect that our findings overestimate the percentages of people with virological suppression. Our analyses do not account for virological ‘blips’, commonly observed in the clinic [32], and we may be underestimating the true number achieving virological suppression. We have no evidence that the frequency of ‘blips’ would vary between countries. On the other hand, our cut-off for virological suppression was high (500 copies/mL), which should reduce the effect of this bias.

Some major strengths of this study should be mentioned. Firstly, we had access to data from a large number of countries, including some with no national data collection structure. Secondly, and in contrast to previous studies [2,19,20], we were able to compare data directly across countries as data was collected in a uniform manner in all countries and across all three time periods. Previous studies have not been able to describe temporal trends, largely due to insufficient follow-up and changes in definitions over time [33]. Finally, we had access to complete data on ART coverage among those followed in EuroSIDA, which is not often readily available in registry studies.

A limitation of our study is that data were based solely on clinics contributing to the EuroSIDA study, which may not be representative of nation-wide care. EuroSIDA is an open cohort and therefore, differences over time may also reflect recruitment patterns over time. Although not a recommended standard treatment strategy in current guidelines [26,27], switching ART in stable patients with undetectable viral load to dual therapy regimens has gained some ground in recent years [26,34,35]. Our definition of ART does not capture patients on such regimens, which may lead to an underestimation of ART coverage. Also, we did not take eligibility for ART into consideration when describing trends in ART uptake over time. In this context it is important to underline that this study did not aim to evaluate adherence to guidelines. Furthermore, it is worth keeping in mind that, while guidelines for when to start ART have changed over time, the goal of virological suppression among those on ART has not.

Partly due to our study design, we used the definition ‘on ART among those in care’ rather than among those diagnosed. Compared with the UNAIDS 90–90–90 definition of ‘on ART’ [18], we expect that our estimates of ART coverage are higher, as the denominator includes only those in care, and the true between-country variation in ART coverage may thus be even more pronounced than what we observed. On the other hand, our definition allowed us to directly compare performance at the clinic level without considering the impact of variation in linkage to care.

Between-country variation in HIV treatment and care is likely to reflect a complexity of underlying reasons including differences in patient populations, patient management, healthcare structures, policies, health expenditure, varying local treatment guidelines and more. In addition to presenting the unadjusted, country-specific estimates that are usually presented in cascades of care, we evaluated the odds of receiving ART and being virologically suppressed on ART across regions. In these analyses, we chose to adjust for basic patient characteristics that were expected to vary across regions and over time. One advantage of the unadjusted snapshot approach is that the estimates are easy to interpret and to communicate, e.g. to policymakers. However, we believe that the adjusted analyses add to the understanding of how much of the regional variation may be attributed to differences in patient characteristics and how much may be attributable to differences in the quality of care, which could be targeted in health policy interventions. For example, in unadjusted estimates, people in Eastern Europe were less likely than people in Western Europe to receive ART. However, after adjustment, the odds of receiving ART were higher in Eastern Europe, indicating that some of the differences in ART coverage in the two regions may be explained by differences in patient characteristics. Needless to say, our analyses are only one step towards understanding the complex interplay of factors that lead to variation. However, we believe that our study may help identify potential gaps in care and may help frame new questions that will give us a better understanding of the causes of variation in the quality of HIV care. One gap in care within the individual regions has been identified for people who inject drugs [36]. In this context, our findings emphasise the need to continue monitoring the response to the HIV epidemic and to construct high-quality data collection that may serve as a platform for both local monitoring and international comparisons.

## Conclusion

We were able to directly compare data from a large number of clinics across Europe, including some countries that do not have national registries. We found persistent between-country disparities in the level of ART coverage and virological suppression, as well as the rate of improvement over the last decade. EuroSIDA will continue the surveillance of changes and variation in countries’ performance in the ‘test and treat’ era. Current EuroSIDA work aims to explore the underlying reasons for the observed variation, with the goal to identify a best practice and to benchmark HIV care.
